# 5-Bromo-2-(4-fluoro­phen­yl)-3-phenyl­sulfinyl-1-benzofuran

**DOI:** 10.1107/S1600536811032387

**Published:** 2011-08-17

**Authors:** Pil Ja Seo, Hong Dae Choi, Byeng Wha Son, Uk Lee

**Affiliations:** a Department of Chemistry, Dongeui University, San 24 Kaya-dong Busanjin-gu, Busan 614-714, Republic of Korea; b Department of Chemistry, Pukyong National University, 599-1 Daeyeon 3-dong, Nam-gu, Busan 608-737, Republic of Korea

## Abstract

In the title compound, C_20_H_12_BrFO_2_S, the 4-fluoro­phenyl ring makes a dihedral angle of 2.63 (6)° with the mean plane of the benzofuran fragment. The dihedral angle between the phenyl ring and the mean plane of the benzofuran fragment is 84.60 (6)°. In the crystal, mol­ecules are linked by weak inter­molecular C—H⋯O hydrogen bonds, and slipped π–π inter­actions between the benzene rings of neighbouring mol­ecules [centroid–centroid distance = 3.719 (3) Å, inter­planar distance = 3.000 (3) Å and slippage = 1.520 (3) Å].

## Related literature

For the pharmacological activity of benzofuran compounds, see: Aslam *et al.* (2009 ▶); Galal *et al.* (2009 ▶); Khan *et al.* (2005 ▶). For natural products with benzofuran rings, see: Akgul & Anil (2003 ▶); Soekamto *et al.* (2003 ▶). For structural studies of related 5-halo-2-(4-halophen­yl)-3-phenyl­sulfinyl-1-benzofuran deriv­atives, see: Choi *et al.* (2010 ▶, 2011 ▶).
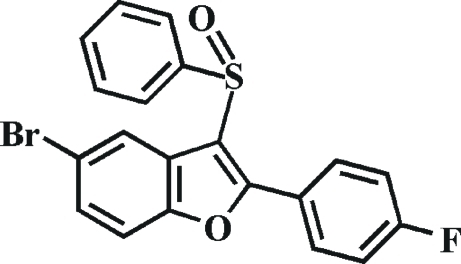


## Experimental

### 

#### Crystal data

C_20_H_12_BrFO_2_S
*M* *_r_* = 415.27
Triclinic, 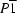

*a* = 8.0090 (2) Å
*b* = 9.8607 (3) Å
*c* = 11.7209 (3) Å
α = 70.471 (2)°
β = 83.171 (2)°
γ = 69.583 (2)°
*V* = 817.59 (4) Å^3^
*Z* = 2
Mo *K*α radiation
μ = 2.66 mm^−1^
*T* = 173 K
0.29 × 0.19 × 0.18 mm

#### Data collection

Bruker SMART APEXII CCD diffractometer
Absorption correction: multi-scan (*SADABS*; Bruker, 2009 ▶) *T*_min_ = 0.513, *T*_max_ = 0.649
15330 measured reflections
4102 independent reflections
3428 reflections with *I* > 2σ(*I*)
*R*_int_ = 0.039

#### Refinement

*R*[*F* ^2^ > 2σ(*F* ^2^)] = 0.032
*wR*(*F* ^2^) = 0.082
*S* = 1.02
4102 reflections
226 parameters
H-atom parameters constrained
Δρ_max_ = 0.34 e Å^−3^
Δρ_min_ = −0.35 e Å^−3^

### 

Data collection: *APEX2* (Bruker, 2009 ▶); cell refinement: *SAINT* (Bruker, 2009 ▶); data reduction: *SAINT*; program(s) used to solve structure: *SHELXS97* (Sheldrick, 2008 ▶); program(s) used to refine structure: *SHELXL97* (Sheldrick, 2008 ▶); molecular graphics: *ORTEP-3* (Farrugia, 1997 ▶) and *DIAMOND* (Brandenburg, 1998 ▶); software used to prepare material for publication: *SHELXL97*.

## Supplementary Material

Crystal structure: contains datablock(s) global, I. DOI: 10.1107/S1600536811032387/zl2399sup1.cif

Structure factors: contains datablock(s) I. DOI: 10.1107/S1600536811032387/zl2399Isup2.hkl

Supplementary material file. DOI: 10.1107/S1600536811032387/zl2399Isup3.cml

Additional supplementary materials:  crystallographic information; 3D view; checkCIF report

## Figures and Tables

**Table 1:** Hydrogen-bond geometry (Å, °)

*D*—H⋯*A*	*D*—H	H⋯*A*	*D*⋯*A*	*D*—H⋯*A*
C13—H13⋯O2^i^	0.95	2.58	3.460 (2)	154
C19—H19⋯O2^ii^	0.95	2.55	3.413 (3)	150

Symmetry codes: (i) 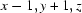; (ii) 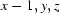.

**Fig. 1.**
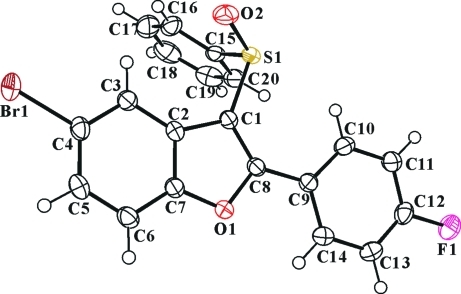
The molecular structure of the title compound with the atom numbering scheme. Displacement ellipsoids are drawn at the 50% probability level. H atoms are presented as small spheres of arbitrary radius.

**Fig. 2.**
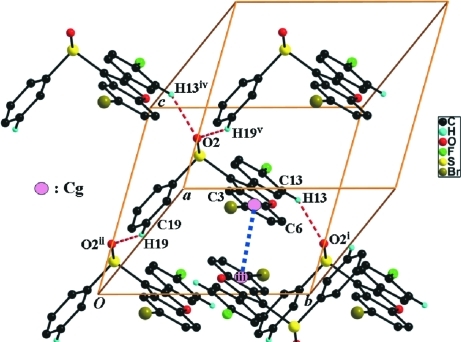
A view of the C—H···O and π–π interactions (dotted lines) in the crystal structure of the title compound. [Symmetry codes: (i) x - 1, y + 1, z; (ii) x - 1, y, z; (iii) - x + 1, - y + 1, - z; (iv) x + 1, y - 1, z; (v) x + 1, y, z.]

## supplementary crystallographic information

### Comment

Many compounds containing a benzofuran ring system have drawn much attention owing to their valuable pharmacological properties such as antibacterial and antifungal, antitumor and antiviral, and antimicrobial activities (Aslam *et al.*, 2009, Galal *et al.*, 2009, Khan *et al.*, 2005). These benzofuran derivatives occur in a wide range of natural products (Akgul & Anil, 2003; Soekamto *et al.*, 2003). As a part of our ongoing study of the substituent effect on the solid state structures of 5-halo-2-(4-halophenyl)-3-phenylsulfinyl-1-benzofuran analogues (Choi *et al.*, 2010, 2011), we report herein the crystal structure of the title compound.

In the title molecule (Fig. 1), the benzofuran unit is essentially planar, with a mean deviation of 0.008 (2) Å from the least-squares plane defined by the nine constituent atoms. The dihedral angle formed by the 4-fluorophenyl ring and the mean plane of the benzofuran fragment is 2.63 (6)°, and the dihedral angle between the phenyl ring and the mean plane of the benzofuran fragment is 84.60 (6)°. The crystal packing (Fig. 2) is stabilized by weak intermolecular C—H···O hydrogen bonds; the first one between a 4-fluorophenyl H atom and the O atom of the sulfinyl group (Table 1; C13—H13···O2^i^), and the second one between a phenyl H atom and the O atom of the sulfinyl group (Table 1; C19—H19···O2^ii^). The crystal packing (Fig. 2) is further stabilized by weak slipped π–π interactions between the benzene  rings of adjacent molecules, with Cg···Cg^iii^ distance of 3.719 (3) Å and an interplanar distance of 3.000 (3) Å resulting in a slippage of 1.520 (3) Å (Cg is the centroids of the C2–C7 benzene ring).

### Experimental

77% 3-chloroperoxybenzoic acid (202 mg, 0.9 mmol) was added in small portions to a stirred solution of 5-bromo-2-(4-fluorophenyl)-3-phenylsulfanyl-1-benzofuran (319 mg, 0.8 mmol) in dichloromethane (30 mL) at 273 K. After being stirred at room temperature for 4h, the mixture was washed with saturated sodium bicarbonate solution and the organic layer was separated, dried over magnesium sulfate, filtered and concentrated at reduced pressure. The residue was purified by column chromatography (hexane–ethyl acetate, 2:1 v/v) to afford the title compound as a colorless solid [yield 67%, m.p. 473–474 K; Rf = 0.70 (hexane–ethyl acetate, 2:1 v/v)]. Single crystals suitable for X-ray diffraction were prepared by slow evaporation of a solution of the title compound in benzene at room temperature.

### Refinement

All H atoms were positioned geometrically and refined using a riding model, with C—H = 0.95 Å for aryl H atoms. *U*_iso_(H) = 1.2*U*_eq_(C) for aryl H atoms.

### Crystal data

**Table:**

C_20_H_12_BrFO_2_S	*Z* = 2
*M**_r_* = 415.27	*F*(000) = 416
Triclinic, *P*1	*D*_x_ = 1.687 Mg m^−^^3^
Hall symbol: -P 1	Mo *K*α radiation, λ = 0.71073 Å
*a* = 8.0090 (2) Å	Cell parameters from 5582 reflections
*b* = 9.8607 (3) Å	θ = 2.3–28.3°
*c* = 11.7209 (3) Å	µ = 2.66 mm^−^^1^
α = 70.471 (2)°	*T* = 173 K
β = 83.171 (2)°	Block, colourless
γ = 69.583 (2)°	0.29 × 0.19 × 0.18 mm
*V* = 817.59 (4) Å^3^	

### Data collection

**Table:**

Bruker SMART APEXII CCD diffractometer	4102 independent reflections
Radiation source: rotating anode	3428 reflections with *I* > 2σ(*I*)
graphite multilayer	*R*_int_ = 0.039
Detector resolution: 10.0 pixels mm^-1^	θ_max_ = 28.5°, θ_min_ = 1.8°
φ and ω scans	*h* = −10→10
Absorption correction: multi-scan (*SADABS*; Bruker, 2009)	*k* = −13→13
*T*_min_ = 0.513, *T*_max_ = 0.649	*l* = −15→15
15330 measured reflections	

### Refinement

**Table:**

Refinement on *F*^2^	Primary atom site location: structure-invariant direct methods
Least-squares matrix: full	Secondary atom site location: difference Fourier map
*R*[*F*^2^ > 2σ(*F*^2^)] = 0.032	Hydrogen site location: difference Fourier map
*wR*(*F*^2^) = 0.082	H-atom parameters constrained
*S* = 1.02	*w* = 1/[σ^2^(*F*_o_^2^) + (0.0418*P*)^2^ + 0.2246*P*] where *P* = (*F*_o_^2^ + 2*F*_c_^2^)/3
4102 reflections	(Δ/σ)_max_ < 0.001
226 parameters	Δρ_max_ = 0.34 e Å^−^^3^
0 restraints	Δρ_min_ = −0.35 e Å^−^^3^

### Special details

**Table:**

Geometry. All esds (except the esd in the dihedral angle between two l.s. planes) are estimated using the full covariance matrix. The cell esds are taken into account individually in the estimation of esds in distances, angles and torsion angles; correlations between esds in cell parameters are only used when they are defined by crystal symmetry. An approximate (isotropic) treatment of cell esds is used for estimating esds involving l.s. planes.
Refinement. Refinement of F^2^ against ALL reflections. The weighted R-factor wR and goodness of fit S are based on F^2^, conventional R-factors R are based on F, with F set to zero for negative F^2^. The threshold expression of F^2^ > 2sigma(F^2^) is used only for calculating R-factors(gt) etc. and is not relevant to the choice of reflections for refinement. R-factors based on F^2^ are statistically about twice as large as those based on F, and R- factors based on ALL data will be even larger.

### Fractional atomic coordinates and isotropic or equivalent isotropic displacement parameters (Å^2^)

**Table:**

	*x*	*y*	*z*	*U*_iso_*/*U*_eq_	
Br1	1.00885 (3)	0.24519 (3)	−0.107040 (19)	0.03957 (9)	
S1	0.63342 (6)	0.12842 (5)	0.38339 (4)	0.02592 (11)	
F1	0.02314 (18)	0.62109 (15)	0.66048 (12)	0.0443 (3)	
O2	0.8243 (2)	0.04378 (17)	0.36866 (14)	0.0362 (3)	
O1	0.49496 (18)	0.57208 (14)	0.20182 (11)	0.0254 (3)	
C2	0.6785 (2)	0.3543 (2)	0.16790 (16)	0.0229 (4)	
C1	0.5920 (2)	0.3166 (2)	0.28359 (16)	0.0228 (4)	
C3	0.7985 (3)	0.2720 (2)	0.09969 (17)	0.0256 (4)	
H3	0.8443	0.1637	0.1280	0.031*	
C4	0.8479 (3)	0.3543 (2)	−0.01080 (18)	0.0279 (4)	
C5	0.7855 (3)	0.5135 (2)	−0.05501 (18)	0.0301 (4)	
H5	0.8259	0.5651	−0.1311	0.036*	
C6	0.6651 (3)	0.5955 (2)	0.01210 (18)	0.0287 (4)	
H6	0.6194	0.7038	−0.0163	0.034*	
C7	0.6142 (3)	0.5126 (2)	0.12269 (17)	0.0242 (4)	
C8	0.4834 (3)	0.4504 (2)	0.30045 (16)	0.0228 (4)	
C9	0.3606 (3)	0.4920 (2)	0.39482 (17)	0.0237 (4)	
C10	0.3378 (3)	0.3843 (2)	0.50252 (19)	0.0312 (4)	
H10	0.4015	0.2792	0.5148	0.037*	
C11	0.2245 (3)	0.4274 (2)	0.59151 (19)	0.0332 (5)	
H11	0.2097	0.3534	0.6649	0.040*	
C12	0.1332 (3)	0.5797 (2)	0.57210 (19)	0.0301 (4)	
C13	0.1499 (3)	0.6903 (2)	0.4678 (2)	0.0347 (5)	
H13	0.0843	0.7948	0.4563	0.042*	
C14	0.2647 (3)	0.6456 (2)	0.37981 (19)	0.0314 (4)	
H14	0.2791	0.7209	0.3072	0.038*	
C15	0.5071 (3)	0.0727 (2)	0.30151 (17)	0.0259 (4)	
C16	0.5951 (3)	−0.0160 (2)	0.2292 (2)	0.0336 (5)	
H16	0.7214	−0.0485	0.2233	0.040*	
C17	0.4956 (4)	−0.0567 (3)	0.1652 (2)	0.0446 (6)	
H17	0.5534	−0.1162	0.1138	0.054*	
C18	0.3125 (4)	−0.0105 (3)	0.1765 (2)	0.0474 (6)	
H18	0.2446	−0.0366	0.1311	0.057*	
C19	0.2264 (3)	0.0733 (3)	0.2530 (2)	0.0450 (6)	
H19	0.1006	0.1015	0.2622	0.054*	
C20	0.3238 (3)	0.1155 (2)	0.3157 (2)	0.0334 (5)	
H20	0.2660	0.1734	0.3682	0.040*	

### Atomic displacement parameters (Å^2^)

**Table:**

	*U*^11^	*U*^22^	*U*^33^	*U*^12^	*U*^13^	*U*^23^
Br1	0.03834 (14)	0.04542 (15)	0.03048 (13)	−0.01050 (10)	0.01344 (9)	−0.01439 (10)
S1	0.0273 (2)	0.0205 (2)	0.0210 (2)	−0.00211 (18)	0.00143 (19)	−0.00188 (17)
F1	0.0399 (7)	0.0455 (7)	0.0390 (7)	−0.0019 (6)	0.0137 (6)	−0.0202 (6)
O2	0.0258 (7)	0.0315 (7)	0.0387 (8)	0.0015 (6)	−0.0035 (6)	−0.0055 (6)
O1	0.0326 (7)	0.0201 (6)	0.0209 (6)	−0.0079 (5)	0.0021 (6)	−0.0048 (5)
C2	0.0243 (9)	0.0248 (9)	0.0187 (9)	−0.0092 (7)	0.0003 (7)	−0.0047 (7)
C1	0.0236 (9)	0.0226 (9)	0.0195 (9)	−0.0066 (7)	0.0007 (7)	−0.0047 (7)
C3	0.0242 (9)	0.0254 (9)	0.0241 (9)	−0.0065 (7)	0.0007 (8)	−0.0058 (7)
C4	0.0242 (9)	0.0345 (10)	0.0234 (9)	−0.0100 (8)	0.0038 (8)	−0.0080 (8)
C5	0.0331 (11)	0.0353 (11)	0.0220 (9)	−0.0171 (9)	0.0039 (8)	−0.0043 (8)
C6	0.0358 (11)	0.0259 (9)	0.0244 (10)	−0.0147 (8)	0.0007 (8)	−0.0035 (8)
C7	0.0268 (9)	0.0245 (9)	0.0214 (9)	−0.0097 (7)	−0.0001 (8)	−0.0061 (7)
C8	0.0280 (9)	0.0209 (8)	0.0190 (9)	−0.0091 (7)	−0.0012 (7)	−0.0040 (7)
C9	0.0235 (9)	0.0239 (9)	0.0226 (9)	−0.0059 (7)	−0.0022 (7)	−0.0073 (7)
C10	0.0352 (11)	0.0223 (9)	0.0309 (11)	−0.0052 (8)	0.0064 (9)	−0.0086 (8)
C11	0.0368 (11)	0.0292 (10)	0.0263 (10)	−0.0067 (9)	0.0069 (9)	−0.0063 (8)
C12	0.0240 (10)	0.0361 (11)	0.0297 (10)	−0.0047 (8)	0.0033 (8)	−0.0162 (9)
C13	0.0339 (11)	0.0261 (10)	0.0366 (12)	0.0006 (8)	0.0005 (9)	−0.0119 (9)
C14	0.0350 (11)	0.0233 (9)	0.0286 (10)	−0.0035 (8)	−0.0002 (9)	−0.0055 (8)
C15	0.0275 (10)	0.0174 (8)	0.0254 (10)	−0.0046 (7)	0.0025 (8)	−0.0013 (7)
C16	0.0375 (12)	0.0239 (9)	0.0374 (12)	−0.0103 (9)	0.0101 (9)	−0.0105 (9)
C17	0.0620 (17)	0.0308 (11)	0.0455 (14)	−0.0202 (11)	0.0068 (12)	−0.0150 (10)
C18	0.0621 (17)	0.0348 (12)	0.0478 (15)	−0.0278 (12)	−0.0128 (13)	0.0004 (11)
C19	0.0351 (12)	0.0371 (12)	0.0531 (15)	−0.0134 (10)	−0.0069 (11)	0.0017 (11)
C20	0.0294 (10)	0.0271 (10)	0.0347 (11)	−0.0054 (8)	0.0046 (9)	−0.0042 (9)

### Geometric parameters (Å, °)

**Table:**

Br1—C4	1.895 (2)	C9—C14	1.399 (3)
S1—O2	1.4852 (15)	C10—C11	1.377 (3)
S1—C1	1.7698 (18)	C10—H10	0.9500
S1—C15	1.794 (2)	C11—C12	1.373 (3)
F1—C12	1.353 (2)	C11—H11	0.9500
O1—C7	1.371 (2)	C12—C13	1.369 (3)
O1—C8	1.382 (2)	C13—C14	1.378 (3)
C2—C3	1.389 (3)	C13—H13	0.9500
C2—C7	1.391 (3)	C14—H14	0.9500
C2—C1	1.441 (3)	C15—C16	1.380 (3)
C1—C8	1.368 (3)	C15—C20	1.385 (3)
C3—C4	1.376 (3)	C16—C17	1.387 (3)
C3—H3	0.9500	C16—H16	0.9500
C4—C5	1.397 (3)	C17—C18	1.380 (4)
C5—C6	1.380 (3)	C17—H17	0.9500
C5—H5	0.9500	C18—C19	1.383 (4)
C6—C7	1.383 (3)	C18—H18	0.9500
C6—H6	0.9500	C19—C20	1.375 (3)
C8—C9	1.457 (3)	C19—H19	0.9500
C9—C10	1.392 (3)	C20—H20	0.9500
			
O2—S1—C1	106.67 (9)	C11—C10—H10	119.5
O2—S1—C15	106.92 (9)	C9—C10—H10	119.5
C1—S1—C15	96.40 (9)	C12—C11—C10	118.66 (19)
C7—O1—C8	106.92 (14)	C12—C11—H11	120.7
C3—C2—C7	119.62 (17)	C10—C11—H11	120.7
C3—C2—C1	135.37 (17)	F1—C12—C13	119.07 (18)
C7—C2—C1	105.00 (16)	F1—C12—C11	118.23 (18)
C8—C1—C2	107.45 (16)	C13—C12—C11	122.70 (19)
C8—C1—S1	128.60 (15)	C12—C13—C14	118.04 (19)
C2—C1—S1	123.90 (14)	C12—C13—H13	121.0
C4—C3—C2	116.94 (18)	C14—C13—H13	121.0
C4—C3—H3	121.5	C13—C14—C9	121.58 (19)
C2—C3—H3	121.5	C13—C14—H14	119.2
C3—C4—C5	123.30 (19)	C9—C14—H14	119.2
C3—C4—Br1	117.97 (15)	C16—C15—C20	121.5 (2)
C5—C4—Br1	118.73 (15)	C16—C15—S1	119.32 (16)
C6—C5—C4	119.88 (18)	C20—C15—S1	119.10 (16)
C6—C5—H5	120.1	C15—C16—C17	118.7 (2)
C4—C5—H5	120.1	C15—C16—H16	120.6
C5—C6—C7	116.77 (18)	C17—C16—H16	120.6
C5—C6—H6	121.6	C18—C17—C16	119.8 (2)
C7—C6—H6	121.6	C18—C17—H17	120.1
O1—C7—C6	125.82 (17)	C16—C17—H17	120.1
O1—C7—C2	110.70 (16)	C17—C18—C19	120.9 (2)
C6—C7—C2	123.48 (18)	C17—C18—H18	119.6
C1—C8—O1	109.92 (16)	C19—C18—H18	119.6
C1—C8—C9	135.27 (17)	C20—C19—C18	119.7 (2)
O1—C8—C9	114.80 (15)	C20—C19—H19	120.2
C10—C9—C14	117.93 (18)	C18—C19—H19	120.2
C10—C9—C8	122.43 (17)	C19—C20—C15	119.3 (2)
C14—C9—C8	119.61 (17)	C19—C20—H20	120.4
C11—C10—C9	121.09 (18)	C15—C20—H20	120.4
			
C3—C2—C1—C8	−178.7 (2)	C7—O1—C8—C9	−179.65 (16)
C7—C2—C1—C8	0.2 (2)	C1—C8—C9—C10	3.7 (4)
C3—C2—C1—S1	3.7 (3)	O1—C8—C9—C10	−177.40 (18)
C7—C2—C1—S1	−177.34 (14)	C1—C8—C9—C14	−178.2 (2)
O2—S1—C1—C8	−143.48 (18)	O1—C8—C9—C14	0.7 (3)
C15—S1—C1—C8	106.68 (19)	C14—C9—C10—C11	0.0 (3)
O2—S1—C1—C2	33.56 (19)	C8—C9—C10—C11	178.1 (2)
C15—S1—C1—C2	−76.27 (17)	C9—C10—C11—C12	0.1 (3)
C7—C2—C3—C4	0.3 (3)	C10—C11—C12—F1	−179.5 (2)
C1—C2—C3—C4	179.1 (2)	C10—C11—C12—C13	0.2 (3)
C2—C3—C4—C5	0.9 (3)	F1—C12—C13—C14	179.0 (2)
C2—C3—C4—Br1	−179.14 (14)	C11—C12—C13—C14	−0.6 (3)
C3—C4—C5—C6	−1.5 (3)	C12—C13—C14—C9	0.8 (3)
Br1—C4—C5—C6	178.56 (16)	C10—C9—C14—C13	−0.5 (3)
C4—C5—C6—C7	0.8 (3)	C8—C9—C14—C13	−178.7 (2)
C8—O1—C7—C6	−179.84 (19)	O2—S1—C15—C16	−8.11 (18)
C8—O1—C7—C2	0.7 (2)	C1—S1—C15—C16	101.52 (16)
C5—C6—C7—O1	−179.09 (19)	O2—S1—C15—C20	169.70 (15)
C5—C6—C7—C2	0.3 (3)	C1—S1—C15—C20	−80.67 (16)
C3—C2—C7—O1	178.60 (17)	C20—C15—C16—C17	3.0 (3)
C1—C2—C7—O1	−0.6 (2)	S1—C15—C16—C17	−179.24 (16)
C3—C2—C7—C6	−0.9 (3)	C15—C16—C17—C18	−1.1 (3)
C1—C2—C7—C6	179.93 (19)	C16—C17—C18—C19	−1.5 (3)
C2—C1—C8—O1	0.2 (2)	C17—C18—C19—C20	2.2 (3)
S1—C1—C8—O1	177.59 (14)	C18—C19—C20—C15	−0.3 (3)
C2—C1—C8—C9	179.1 (2)	C16—C15—C20—C19	−2.3 (3)
S1—C1—C8—C9	−3.5 (4)	S1—C15—C20—C19	179.90 (16)
C7—O1—C8—C1	−0.5 (2)		

### Hydrogen-bond geometry (Å, °)

**Table:**

*D*—H···*A*	*D*—H	H···*A*	*D*···*A*	*D*—H···*A*
C13—H13···O2^i^	0.95	2.58	3.460 (2)	154.
C19—H19···O2^ii^	0.95	2.55	3.413 (3)	150.

Symmetry codes: (i) *x*−1, *y*+1, *z*; (ii) *x*−1, *y*, *z*.
